# Neural network model applied to electromagnetic shielding effectiveness of ultra-light Ni/Cu coated polyester fibrous materials

**DOI:** 10.1038/s41598-022-12593-8

**Published:** 2022-05-21

**Authors:** Aravin Prince Periyasamy, Lekha Priya Muthusamy, Jiri Militký

**Affiliations:** 1 Department of Bioproducts and Biosystems, School of Chemical Engineering, Aalto University, Espoo, Finland; 2 Department of Mathematics, Government Arts College, Coimbatore, India; 3 Department of Material Engineering, Faculty of Textile Engineering, Technical University of Liberec, Studentska 2, Liberec, 46117 Czech Republic

**Keywords:** Chemistry, Materials science, Mathematics and computing

## Abstract

The purpose of effective electromagnetic interference (EMI) shielding is to prevent EMI from smartphone, wireless, and utilization of other electronic devices. The electrical conductivity of materials strongly influences on the EMI shielding properties. In this work, mainly focus to predict the EMI shielding effectiveness on the ultralight weight fibrous materials by artificial neural network (ANN). Prior to the ANN modelling, the ultra-lightweight fibrous materials were electroplated with different concentration of Ni/Cu and then coated with different silanes. This work utilizes the algorithm to provide accurate quantitative values of EMI shielding effectiveness (EM SE). To compare its performance, the experimental and the predicted EM SE values were validated by root-mean-square error (RMSE), mean absolute percentage error (MAPE) values and correlation coefficient ‘r’. The proposed ANN results accurately predict the experimental data with correlation coefficients of 0.991 and 0.997. Further due to its simplicity, reliability as well as its efficient computational capability the proposed ANN model permits relatively fast, cost effective and objective estimates to be made of serving in this industry.

## Introduction

Electromagnetic pollution is a pervasive environmental health hazard caused by the utilization of various smart devices, and it poses a direct threat to people’s quality of life and health^1^. In general, most electronic devices release electromagnetic radiation at various frequencies, which when exposed to the human body can cause major health problems, therefore, it is significantly important to shield the EMI^1–4^. Furthermore, when electrical equipment is subjected to EMI, they may fail to perform correctly, and these failures might have a significant influence on extremely sensitive electronic devices utilized in civic, commercial, military, or other advanced technical domains^5^. As a matter of fact, the primary goal of EMI shielding is to protect humans as well as electronic equipment by preventing EMI from affecting sensitive electronics. Shielding of electromagnetic materials protect EM waves, which could be achieved through the absorption and reflection of EM radiation.

Metals are good electrical conductors, capable of absorbing, reflecting, and transmitting electromagnetic waves. Earlier days, textile based electromagnetic shields are made of metallic wires (metallic fibers). Metallic coated textiles are classified into protective textiles due to their multi-functionality^6,7^ lightness, flexibility, comfort, better mechanical properties which replace the conventional metallic materials in the electromagnetic shielded textiles^8,9^, in addition the metal deposited fabrics shows multifunctionality effects such as antimicrobial^10^, antiviral^6,7^ properties. There are different techniques involving depositing the metal particles in the fabric materials, electroless plating is one of the popular method due to the efficient with better fabric appearance. Perhaps, metals are usually rigid, heavy, costly and gets oxidation/corrosion easily.

The major disadvantage of metalized fabric is their durability as well as subsequent chemical reactions. Since, copper has possibility to undergo the oxidation^11^. In this work, attempts have been made to produce ultra-light Ni/Cu coated polyester fibrous materials with different concentration by using of electroless plating method. Later, we tried to stabilize the metal coated fabric by simple sol–gel coating. In general, the precursor has a twofold purpose on the Ni/Cu coated polyester fibrous material as it stabilizes the Ni/Cu particles on the fabric’s surface, and it has a major impact on the electromagnetic shielding.

The substitute to traditional analytical methods used widely in analyzing data as an artificial intelligence tool is artificial neural networks (ANNs). ANN has developed widely due to its method in flexible modelling of real-world problems capsulizing the inter-relationships among input and output data pairs that are unknown, nonlinear, or too difficult to formulate, demonstrating its effectiveness in solving difficult and complex engineering problems. The main objective of implanting ANN is being cost-effective, time-effective, and precisely predictive soft model under investigation. ANN comprises of neurons and pathways between them (model of human brain). A set of information is reflected on each neuron as a quantity of variables resulting in output^12^. Because of its technique in flexible modeling of real-world situations encapsulating the inter-relationships among uncertain, nonlinear, or difficult to define input and output data pairs, ANN has grown in popularity, proving its ability in handling challenging and complicated engineering challenges. Main limitation is strong dependence on so called training data set used for creation of ANN (statistically it is so-called nonparametric smoothing being often overparametrized) and no possibility to predict behavior of ANN model out of range of training set.

ANN are applied in various fields like as water resources engineering, traffic engineering^13^, detection of structural damage^14^, concrete strength prediction^15^, in textiles, yarn strength^16,17^, fabric pilling properties^18^, mechanical properties^19^, color strength prediction^20^, fabric finishing^21^, microplastic emission during domestic washing^22^.

In this work, we developed ultra-light weight Ni/Cu coated polyester fibrous materials via electroless plating techniques and subsequently the conductive fabric was coated with different precursor to know their influence on the electromagnetic shielding properties. Aim of this study is to predict the EMI shielding effectiveness (EM SE) of ultra-light weight Ni/Cu coated polyester fibrous materials. Following that, the prediction model was built using an artificial neural network. Experiments were utilized to obtain the training data set then used to create the ANN model. Meanwhile, a neural network model with two hidden layers was built, and it was trained and tested using 36 groups of data from experiments. There were two input parameters and one output parameter in the neural network model. The model's dependability was confirmed by comparing the predicted results to the test outcomes.

## Results and discussion

### Surface characterization

**Figure 1:**
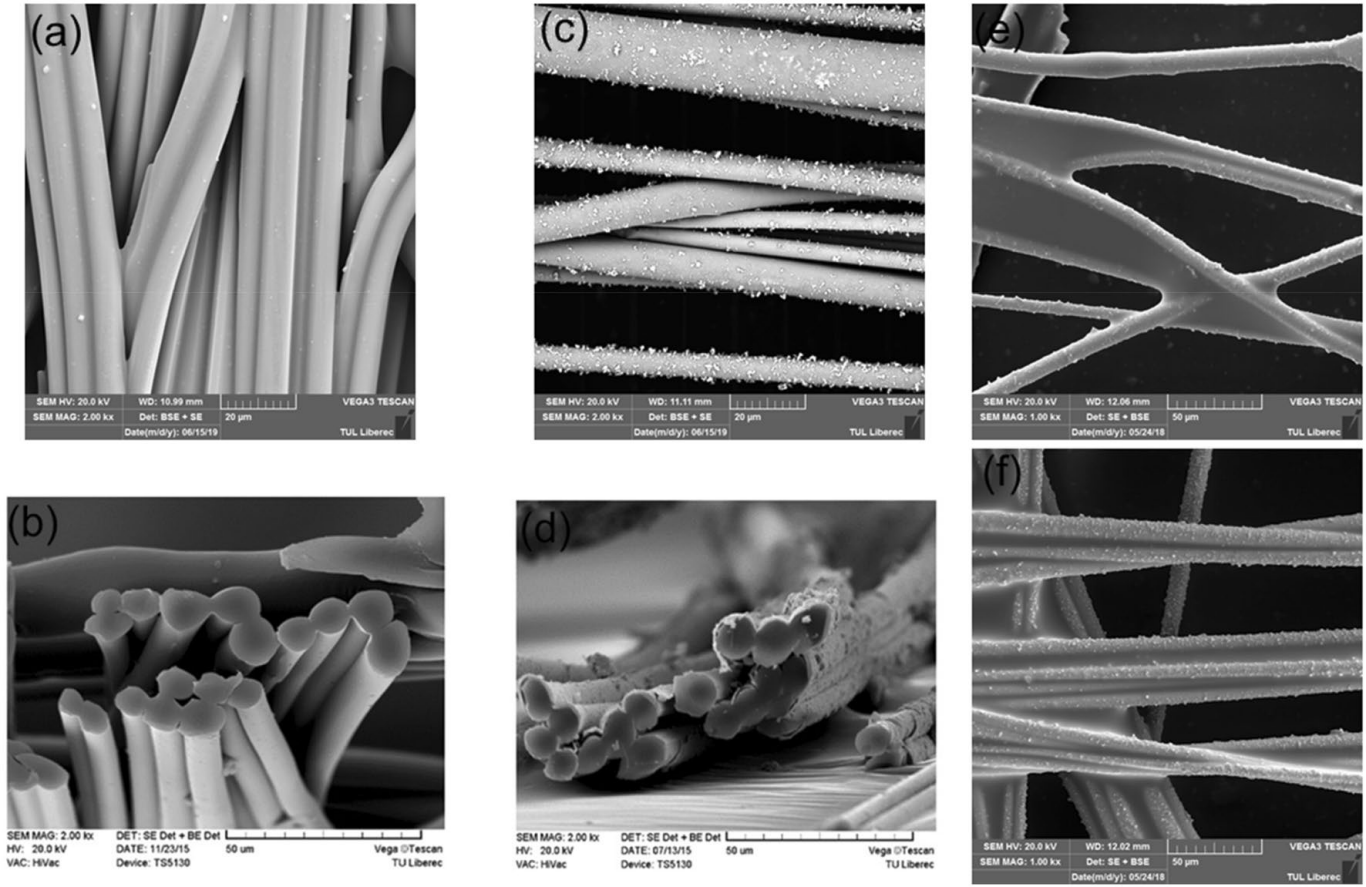
SEM images of (**a**) pristine; (**d**) PET fibrous material with Ni/Cu; cross section of the (**b**) pristine; (**d**) PET fibrous material with Ni/Cu; Ni/Cu deposited PET fabric with OTES (**e**) and PhTES (**f**).

Surface properties of conductive fabric was studied by SEM and the micrographs are shown in Fig. 1. The pristine fibrous shows the plain and doesn’t have any metal deposition (i.e., no surface roughness was observed) Fig. 1a, in contrast, nickel-coated samples show metal particle accumulation on the surface Fig. 1c. Perhaps, as can be seen in Fig. 1c, the surface roughness has grown as the metal concentration has increased. A cross sectional view of pristine (Fig. 1b) and ultra-light weight Ni/Cu coated polyester fibrous materials is shown in (Fig. 1d), from this image it is evidenced that the deposition of Ni/Cu on the fibrous surface. Examining high-resolution SEM pictures in more detail (Fig. 1e,f), the silanes are covered the Ni/Cu deposition on the fiber surface. Perhaps, the silane possesses almost the similar structure feature on the ultra-light weight Ni/Cu coated polyester fibrous materials. The results of fabric density and thickness are listed Table 1, it exhibited a more homogeneous distribution of Ni/Cu particles on the fibrous materials can be validated by the thickness, density and surface morphology of Ni/Cu coated fabric. Figure S1 displays the documented information of EDS from Ni/Cu plated PET fabrics. Strong spectra demonstrated together with certain elements like copper, nickel, titanium, carbon, and oxygen, confirms the presence of various elements.

**Table 1:** Various properties of ultra-light Ni/Cu coated polyester fibrous materials.

Fabric code	NiSO_4_·6H_2_O Content in the bath (wt%)	Total mass of Ni/Cu on surface (g/m^2^)	Ni content in Ni/Cu coating (Ni) (wt%)	Thickness (mm)	Ariel density (g/m^2^)
N0	0	0.572	0	0.097 ± 0.001	9.24 ± 0.12
N1	2	0.574	4.8	0.101 ± 0.001	9.31 ± 0.17
N2	4	0.571	5.3	0.103 ± 0.004	9.45 ± 0.14
N3	6	0.606	7.1	0.103 ± 0.004	9.82 ± 0.19
N4	8	0.577	8.8	0.108 ± 0.012	10.12 ± 0.22
N5	15	0.475	15.2	0.107 ± 0.017	10.68 ± 0.31
N6	20	0.517	16.6	0.112 ± 0.015	10.82 ± 0.28
N7	25	0.522	19.5	0.112 ± 0.013	11.08 ± 0.38

### Thermo gravimetric analysis (TGA)

**Figure 2:**
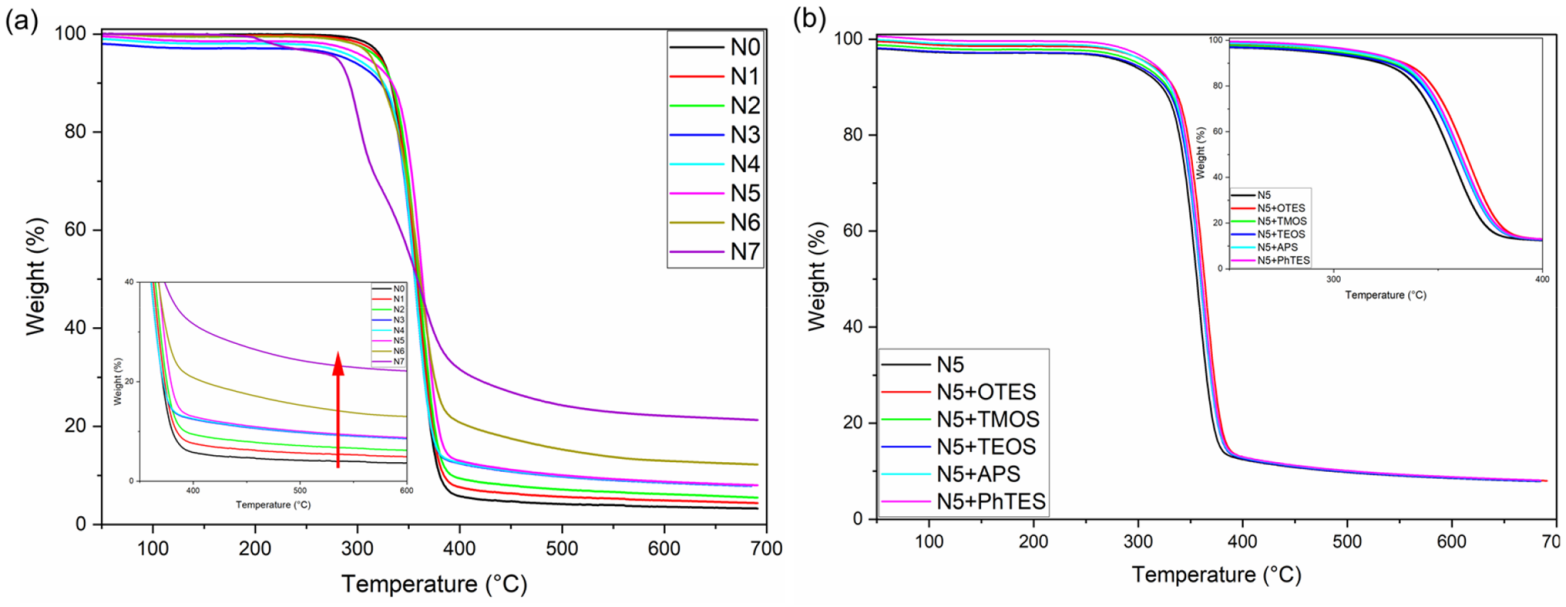
Influence of Ni/Cu concentration (**a**) and sol–gel coating, (**b**) on the ultra-light weight Ni/Cu coated polyester fibrous materials.

The study of thermal stability of ultra-light weight Ni/Cu coated polyester fibrous materials with and without addition of sol–gel treatment is studied by TGA analysis. The increased thermal stability of ultra-light weight Ni/Cu coated polyester fibrous materials fabric is displayed in Fig. 2a and it switches to higher range of temperature rather than the sample without the addition of metal (i.e. N0). There are three steps in the process of principal weight losses in general. Effect of drying temperature for removing water from the organic pigment surface at the temperature range 290–325 °C observed to be the first degradation. This is also, due to the escape of volatile components the first step of the curve occurs. There is no reduction or oxidation in a nitrogen atmosphere. Nevertheless, the degradation of volatile components, water molecules and the volatile functional groups takes place at respective temperatures compared to that of TGA curve of ultra-light weight Ni/Cu coated polyester fibrous materials attained under air atmosphere. Further at 325–375 °C the second degradation is observed for 325–375 °C. The decomposition of polymeric materials (i.e., PET) starting at temperature of 375 to ~ 475 °C contributes the third degradation. Beyond 410 °C, it is observed that the curvature reveals crucial gradual stability with a residual constant mass for Ni/Cu predominately indicating the thermal stability of ultra-light weight Ni/Cu coated polyester fibrous materials. Around 330 °C, occurrence of main weight loss of ultra-light weight Ni/Cu coated polyester fibrous materials is observed due to extra stable oxygen-carrying functional groups existing in the ultra-light weight Ni/Cu coated polyester fibrous materials.

Figure 2b displays the curvatures of TGA of ultra-light weight Ni/Cu coated polyester fibrous materials with and without sol–gel coating. The initial decomposition temperature of the sol–gel coated ultra-light weight Ni/Cu coated polyester fibrous materials is enhanced from 337 to 400 °C to be observed and further increase of char residue amount is observed to be 12% to 15% during the third part of its pyrolysis. The existence of various functional groups of silanes (R–Si, Si–O–Si, Si–O, Si–C) with their bond energy and surface enhancement features and safeguarding the ultra-light weight Ni/Cu coated polyester fibrous materials when contacted with heat sources to certain extent is evidently proved by these results. Thus, an improvised thermal stability of ultra-light weight Ni/Cu coated polyester fibrous materials with sol–gel coating process was formulated.

### Effect of Ni/Cu concentration and precursor on EMSE

**Figure 3:**
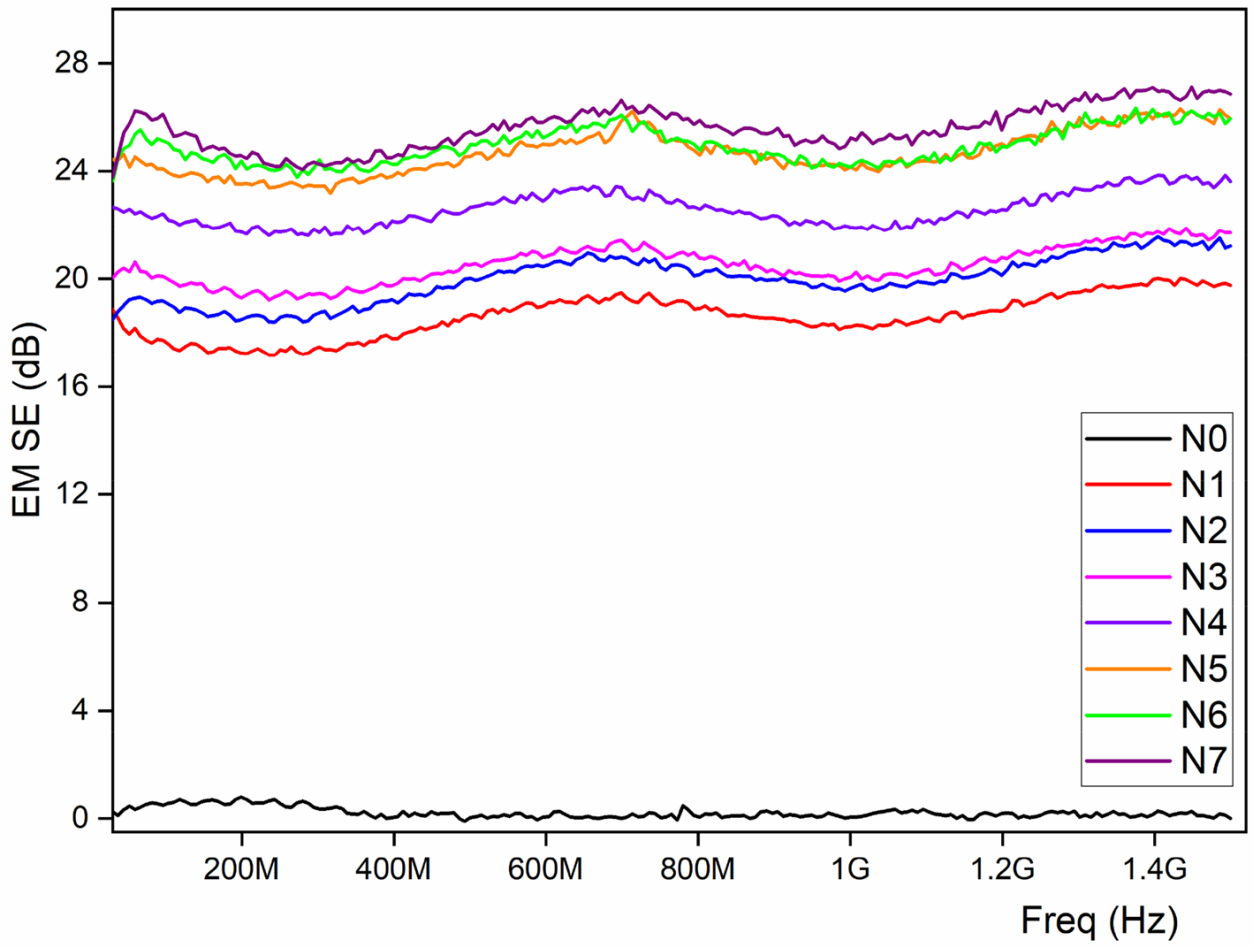
Influence of Ni/Cu concentration on the EM SE.

**Figure 4:**
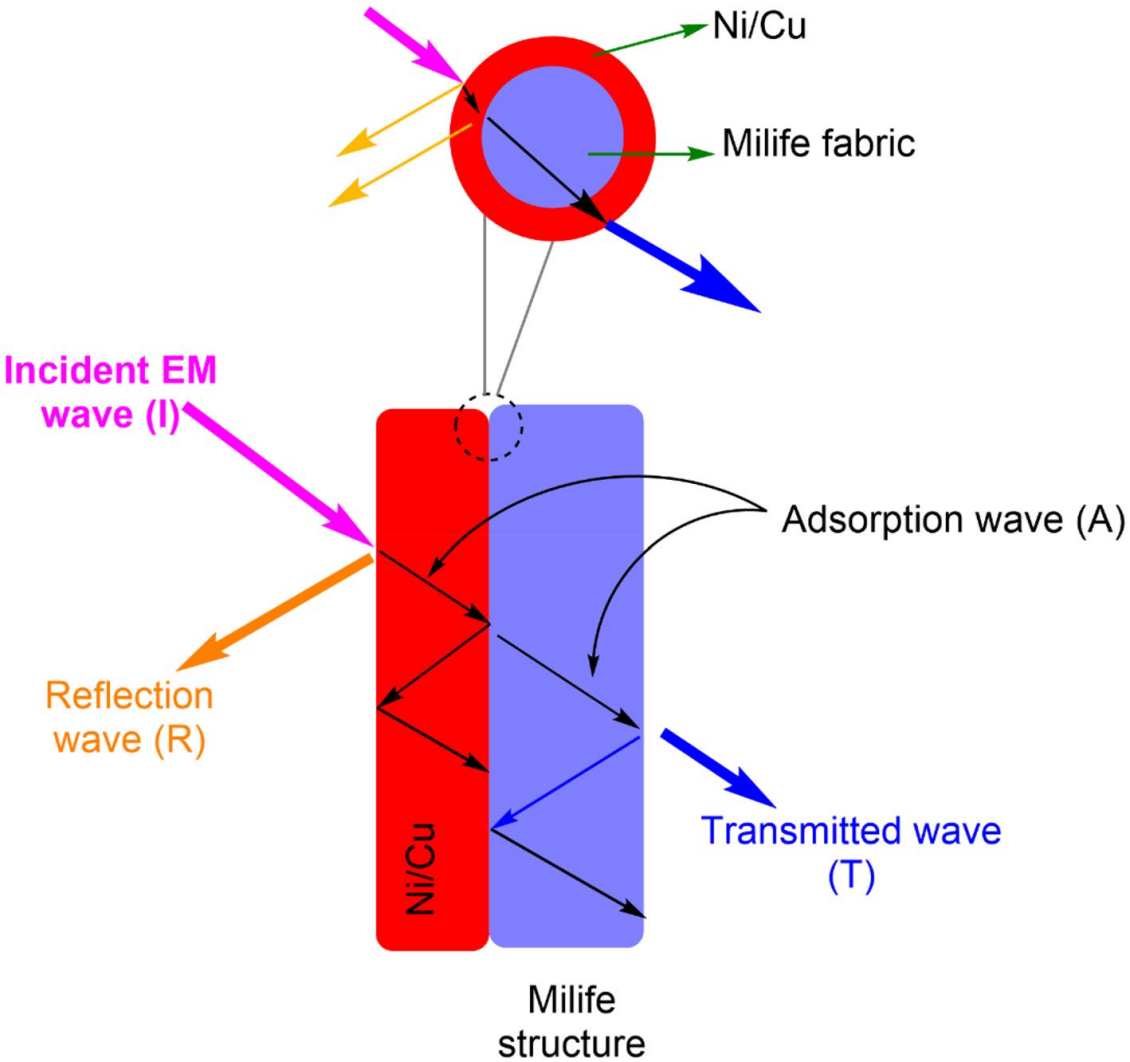
Schematic representation of Ni/Cu coated fibrous materials under EM atmosphere.

Electromagnetic shielding properties are usually depending on the reflection (SE_R_) and absorption (SE_A_), both are directly proportional to the EM SE values, as it is higher is nothing but the higher the electromagnetic shielding properties. Theoretically, shielding efficiency (i.e., SE ~ SE_R_ + SE_A_) mainly depends on the amount of metal deposition on the fibrous surface and the surface activation process, in this work, we have used various concentration of Ni/Cu and the results are proving the theoretical statement. There is no significant improvement after 15% of Ni/Cu concentration as the phenomenon is stable and it provide ~ 24 dB of EM SE until 1.5 GHz (Fig. 3). According to mixed-potential theory, during the initial stage of a low nickel salt concentration local cathode process, a little amount of Ni^2+^ ions can be abridged. The electroless plating efficiency is great even though nickel particles are little absorbed on ultra-light Ni/Cu coated polyester fibrous materials. The adsorption and reflection losses of the incoming EM wave are aided by the magnetic nickel-plated metal. Because of the extremely high concentration of NiSO_4_, which releases an excess of Ni^2+^ ions, the side reactions that form the Ni(OH)_2_ precipitate are activated. The conductive coating on ultra-light weight polyester fibrous materials with the three-dimensional (3D) structure of Ni/Cu conductive network was readily established by this unique architecture. The polyester fibrous materials with Ni/Cu can be considered to consist of numerous core-shelled heterostructures with conductive Ni/Cu coating layers^23,24^. Figure 4 represent the schematically the EMI shielding mechanism of Ni/Cu coated fibrous materials under EM atmosphere. As it will eliminate the chemical balance in the plating solution and overwhelming the chemical reduction reaction. The subsequent fabric might be regarded as “very-good grade” for over-all civil use (such as maternity dress, apron, consumptive electronic products, and communication related products) referring to the report^25^.

#### EM SE on ultra-light Ni/Cu coated polyester fibrous materials at 1.5 GHz frequency

Perhaps, most of the electronic equipment operates at or near this frequency, the EM shielding effectiveness of both Ni/Cu coated, and uncoated cloth was evaluated at 1.5 GHz^26^. Description of textiles requirements with EM shielding is tabulated in (Table 2). Professional and general use are the two types of it. Further it is also categorized into 5 grades from fair to excellent. Table 3 displays the controlled ultra-light Ni/Cu coated polyester fibrous materials < 2 dB. For 25% of Ni/Cu coated polyester fibrous materials delivers EM SE of 26.86 dB at the frequency of 1.5 GHz. Similarly, EM SE of 25.92 was observed with the 15% Ni/Cu, as it confirmed that there is no remarkable change in the EMISE between the concentration of 15 and 25% of Ni/Cu. Nonetheless, EM SE ranges between 20 and 30 dB based on the report^25^, signifying very good grade to implement in general and professional applications.

**Table 2:** Classification of EM SE values on professional and general use^27^.

Usage/grade	Excellent	Very good	Good	Moderate	Fair
Professional use	SE > 60 dB	60 dB ≥ SE > 50 dB	50 dB ≥ SE > 50 dB	40 dB ≥ SE > 30 dB	30 dB ≥ SE > 20 dB
General use	SE > 30 dB	30 dB ≥ SE > 20 dB	20 dB ≥ SE > 10 dB	10 dB ≥ SE > 7 dB	7 dB ≥ SE > 5 dB

**Table 3:** Neural network configuration for the training.

Parameter	Specification
Number of neurons in input layer	2
Number of neurons in hidden layer	10
Number of neurons in output layer	1
Training function	Levenberg–Marquardt (trainlm)
Performance function	Mean square error (MSE)
Activation function	Log-sigmoid

**Figure 5:**
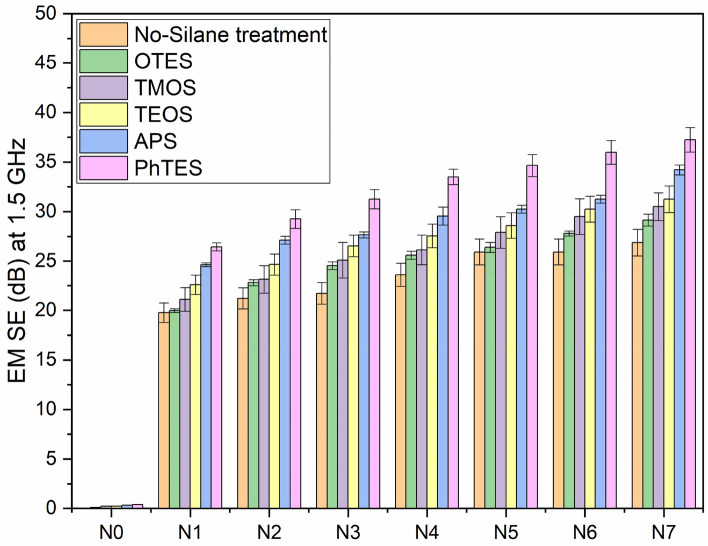
Silinization treatment on EM SE of Ni/Cu coated polyester fibrous materials.

In this work, treatment with silane increase the EM SE, perhaps, it depends on the silane chemistry. Among the silane treatment, PhTES outperforms, as it is containing the aromatic ring in their chemical structure having the conjugated system. In the conjugated system, the ground and excited state of these electron are closer than nonconjugated systems. Therefore, in conjugated systems the electrons are excited with lower energy of EM spectrum. Hence, PhTES absorb even lower energy of EM spectrum is the responsible for the improvement of EM SE values. On the other hand, shielding effectiveness of OTES treated Ni/Cu coated polyester fibrous materials is indirectly proportional to the frequency where linear trend was visible. TEOS treated Ni/Cu coated polyester fibrous materials shows higher electromagnetic shielding effeteness than OTES with Ni/Cu coated polyester fibrous materials, however it is less than the without silanization process, which is described in Fig. 5. In general, alkoxy silanes such as TEOS and OTES have a flexible aliphatic structure that cannot absorb electromagnetic radiation, reducing shielding effectiveness. Generally, the alkoxy silane including TEOS and OTES has the flexible aliphatic structure, which is not capable to absorb the electromagnetic energy, resulting in the reduction of shielding effectiveness.

### Creation of artificial neural network and results

The analysis of EMI shielding effectiveness is formulated by grouping the 2 variables (i.e., Ni/Cu concentrations and silane types) from the experimental attributes. As this process is developed by integrating the input and generating the variables for data learning. Artificial neural network was implemented to analyze the information processing system where this allows to solve the complex problems in classifying, estimating, and optimizing the linear or non-linear relationship between the input and output variables.

Analysis consists of five steps; the first phase is to select technical parameters having a significant impact on the quantity of EMI shielding effectiveness. Secondly, the database has been generated with the factors of EMI shielding effectiveness. The matrices of input and target was created, and further training was established in ANN for simulation. The database was comprised of following variables: concentration of Ni/Cu and sol–gel like OTES, TMOS, TEOS, APS, PhTES were chosen to the input variables. EMI shielding effectiveness values of the fabric was chosen to be the single output for the model.

The database was built where the numerical range presented was in different intervals. This problem was solved by normalizing the variables where the values are in the range [0, 1]. The formula implemented is as follows:

1 $$X_{N} = \frac{{X - X_{\min } }}{{X_{\max } - X_{\min } }}$$

The database was normalized (*X*_*N*_) and generated in the MATLAB. The input matrix corresponds to the concentration of Ni/Cu and target matrix is corresponded to EMI shielding effectiveness. This relationship is linear, and the performance is done row wise. The number of data base utilized for this work is 1440. To enable training and testing of the ANN model, the data source was divided into training and data sets. 70 percent of the data was used for training, and 15 percent of the data was used to test the trained model. 15% of the experimental data generated was used as validation data to evaluate the network and change its settings (such as the number of neurons in the hidden layer). The neural network configuration for the model to be trained and formulating is shown in Table 3.

**Figure 6:**
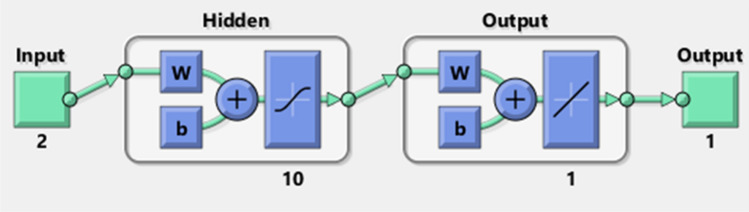
Schematic diagram of the proposed ANN model with hidden layer.

The neural network is established from this relationship in predicting the EMI shielding effectiveness. The training parameters are considered by processing within the ANN model. The back-propagation algorithm presents better performance in this study. This technique uses a variety of designs to identify the best simulation of the input data. Depending on the error the better performed simulation was taken into account. The architecture of the BP algorithm is defined as the selection of parameters and their arrangements in relation to working conditions and modifications to the accuracy of the output data. Figure 6 depicts the schematic diagram of the proposed ANN with hidden layer. The activation function of the first and second layer are log-sigmoid function.

Validation test results prove that the prediction performance of the model more accurately. This leads in selecting the structure of ANN with different parameters. The architectures were Levenberg–Marquardt (LM), scaled conjugate gradient (SCG) and resilient back propagation (BR) which displays better results in estimating of EM SE values. From this structure, 50 architectures were obtained, out of it 12 were taken for simulating. The architecture (2–10–1) refers to as 2 inputs 10 neurons and 1 output, which described in Fig. 9. The results of structures and their performance are tabulated in Table 4.

**Table 4:** MSE of architectures LM, SCG and resilient BR.

Structure of ANN	Algorithm	MSE
2–10–1	LM	0.0912
2–12–1	LM	0.2283
2–15–1	LM	0.1219
2–18–1	LM	0.0299
2–11–1	BR	0.5406
2–13–1	BR	0.3115
2–15–1	BR	0.7205
2–20–1	BR	0.8012
2–10–1	SCG	61.401
2–15–1	SCG	104.9006
2–9–1	SCG	62.1233
2–20–1	SCG	19.1316
2–26–1	SCG	12.8431

**Figure 7:**
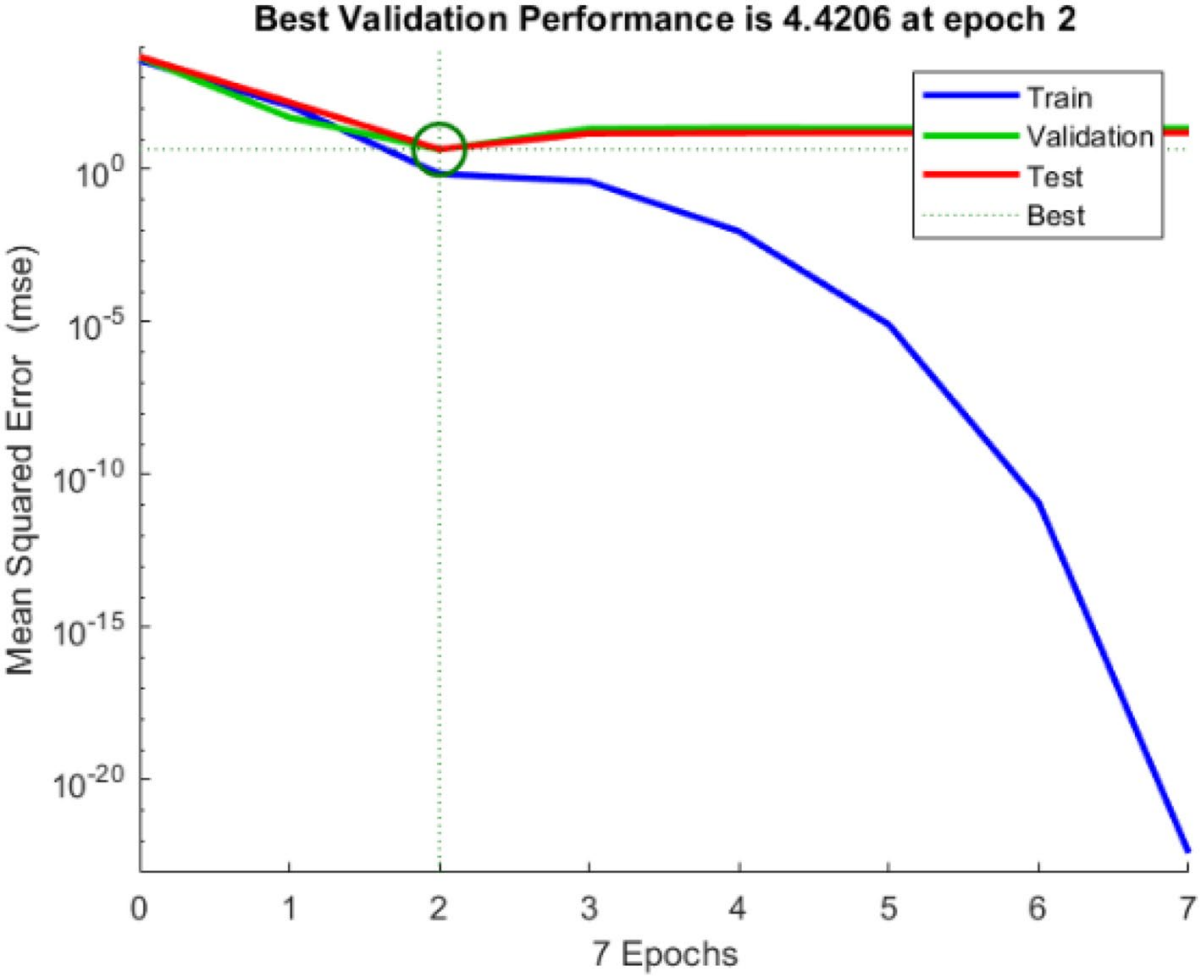
MSE vs epoch for training, validation, and testing sets.

In Table 4, ANN structure of 2 inputs, 15 hidden layers and 1 output was trained under LM structure to calculate the MSE (i.e., 0.1219 < 5), which confirms the forecast values are closer to the experimental value. From analyzing the tabulated results, it shows that the model trained with LM has better performance in predicting the EM SE values with lower MSE. Henceforth, LM was implemented in analyzing the model performance. In order to investigate the network response in more depth, a regression analysis was done between the network output and the corresponding targets (Fig. 7).

In Fig. 7, the training steps of the proposed ANN prediction 2–10–1 structure is shown with concerning epochs. At epoch 2, the best result for validating set is attained further the training is stopped after 7. Figure 7 shows that the MSE was 4.4206 at epoch number 2. The MSE is high in other epochs (epochs number 1 and 2). At epochs 4 and above, the MSE of the model on training data decreases while it increases on validation data. The best model's performance (at epoch 2) was compared to the testing dataset. It is worth noting that model training continues as long as the network's error on the validation vector decreases. Furthermore, the analysis stop point is equivalent to 7, i.e., 6 error repetitions following the epoch with the best validation performance, i.e., epoch 2.

**Figure 8:**
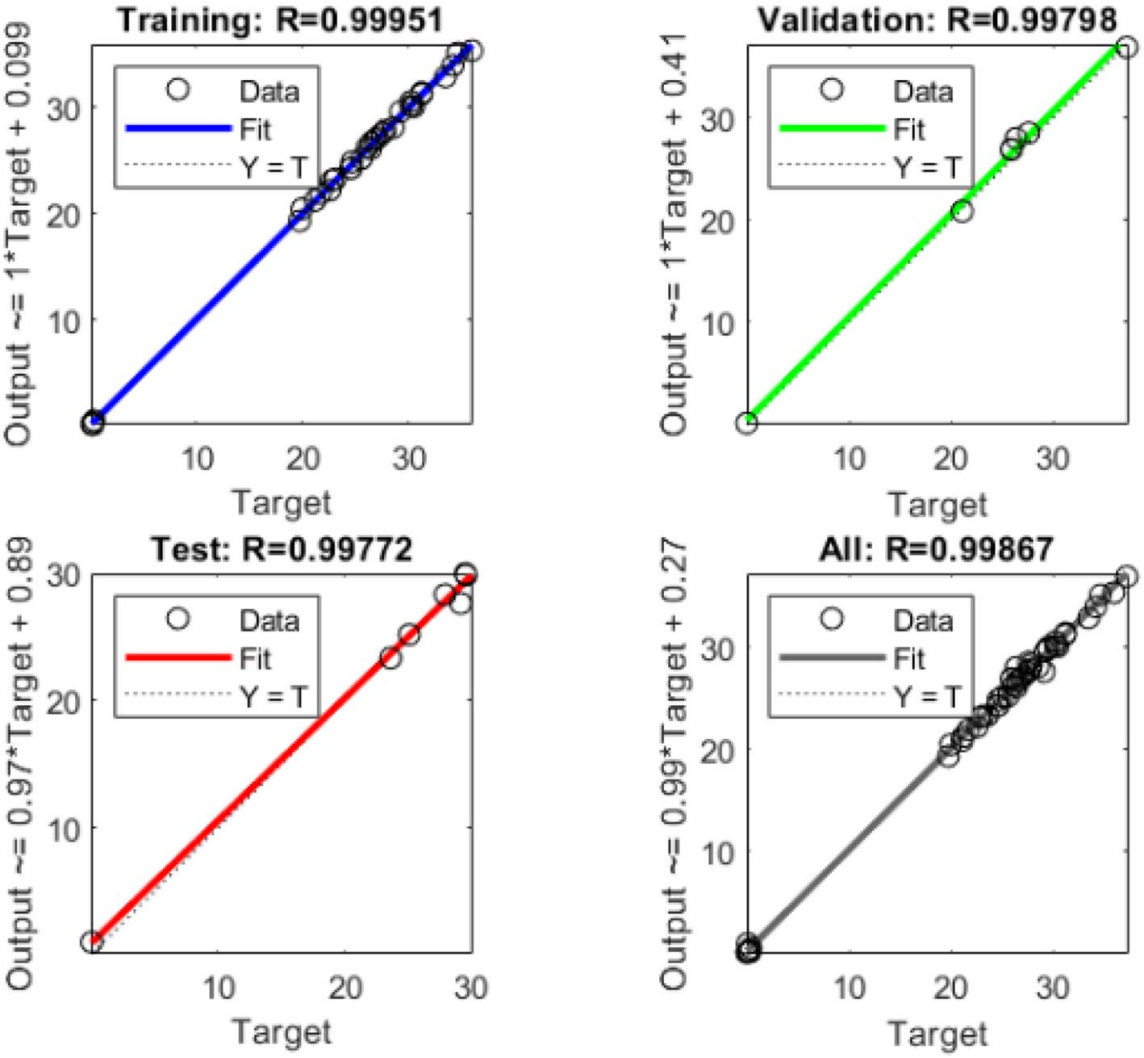
The output of the 2–10–1 ANN concerning the target value for the training, validation, testing and all the data sets.

The ANN anticipated outputs vs experimental values are shown in Fig. 8, which shows the output of the 2–10–1 ANN for the target value for training, validation, testing, and all data sets. The number of neurons in each layer is dictated by the problem’s complexity and data sets. The number of neurons in the hidden layer must be determined in order to build the best model network. It should be mentioned that when the hidden layer has a limited number of neurons, the network’s output training performance is poor. However, increasing the number of neurons beyond 22 has no noticeable effect on network performance. Therefore, we will use 4 to 20 neurons in a hidden layer. To analyze the performance of the neurons, 10, 12, 15, and 18 were picked at random. In this simulation, 300 iterations were used to complete the process of reaching the objective of zero.

Correlation coefficient (r) of predicted and experimental values are analyzed to find the correlation strength and direction of relationship. The correlation coefficient (r) is shows in Table 5 for the EMI shielding prediction model of 4 different structures, as it indicates a strong positive correlation between predicted model and experiment data. Mean absolute percentage error (MAPE) is a measure representing the accuracy a forecast model. Further table displays the correlation between the output of ANN and the targeted value of EM SE. The inconsistency amid the anticipated to the experimental values is given by absolute error whereas it is tending to zero. Moreover, MAPE is made to determine exactly how accurate the forecast values of EM SE values as compared to the experimental values. The mean absolute error (MAPE) amid the experimental value and the predicted values EM SE values was located to be 0.5482% (< 5%) which is lowest values to have the highest accuracy tabulated in Table 5.

**Table 5:** Correlation coefficient and mean absolute error values for ANN structure.

ANN structure	Algorithm	R	MAPE
2–10–1	LM	0.9995	0.5482
2–12–1	LM	0.9998	1.342
2–15–1	LM	0.993	2.346
2–18–1	LM	0.999	0.432

The results demonstrated that the model developed was adequate for predicting EMI shielding. A combination of the previously described performance indicators can offer an unbiased evaluation of the neural network models' prediction abilities. As a result, it is possible to deduce that the suggested ANN design can address up to 99 percent of the overall variability for EMI shielding.

In this work, the ultra-lightweight fibrous materials were electroplated with different concentration of Ni/Cu and then coated with different silanes. The prediction based on the ANN algorithm provides the accurate quantitative prediction on the EM SE values. The results were compared its performance, the experimental and the predicted EM SE values were validated by root-mean-square error (RMSE), mean absolute percentage error (MAPE) values and correlation coefficient ‘r’. The inconsistency amid the anticipated to the experimental values is given by absolute error whereas it is tending to zero. Moreover, MAPE is made to determine exactly how accurate the forecast values of EM SE as compared to the experimental values. The mean absolute error (MAPE) amid the experimental value and the predicted values for the architecture 2–10–1 is 0.5482 and the maximum MAPE was observed 2.346 for the architecture 2–15–1, as it confirmed the architecture with lowest values to have the highest accuracy. The correlation coefficient ‘r’ between the experimental EM SE and that anticipated EM SE by the ANN model is 0.99 (i.e., for all architecture). Therefore, it concluded that the proposed ANN model accuracy up to 99% of the total variability for EM SE values. The uncertainty conditions are effectively handled by the ANN model as it provides support to the decision makers when there is an ambiguity in EM SE measurement. The prediction of EM SE values of fibrous materials is supported by the dynamic decision-making process as it can be implemented through computer systems. Further due to its simplicity, reliability as well as its efficient computational capability the proposed model permits relatively fast, cost effective and objective estimates to be made of serving the EM SE measurement in the industry.

## Materials and methods

### Materials

100% polyester filament cross-laminated composite nonwovens that combines machine direction and cross direction oriented nonwoven layers (ultra-light weight Ni/Cu coated polyester fibrous materials) have been used for this study. The metallization has been well explained in our previous work^2^, physical properties of ultra-light Ni/Cu coated polyester fibrous materials are shown in Table 1. For sol–gel coating, different precursors were used namely, Triethoxyvinylsilane (TVS), Triethoxyphenylsilane (PhTES), Vinyltrimethoxysilane (VTMS), Tetramethyl orthosilicate (TMOS) and Tetraethyl orthosilicate (TEOS) were purchased from Sigma Aldrich Germany. All the chemicals are in the analytical grade and used in our work as we received.

### Sol–gel synthesis

For silanization treatment on the conductive fabric, the following precursors like OTES, TMOS, TEOS, APS, PhTES were used. The sol–gel synthesis was done by mixing of precursors with catalyst, solvent, and water with 1:4:8 respectively^28–30^, later the mixture was adjusted the pH of 3 by using of 0.1 M of HNO_3_. Later water added slowly (1 mL/h) with the help of metering pump; therefore, the precursors have time to react slowly to form sols. After 8 h, prepared sol has been coated on the conductive fabric by simple dip coating. After dip coating, the conductive fabric allowed to dry in the atmospheric air and then it cured at 150 °C for 3 min in the stenter.

### Neural network modelling

**Figure 9:**
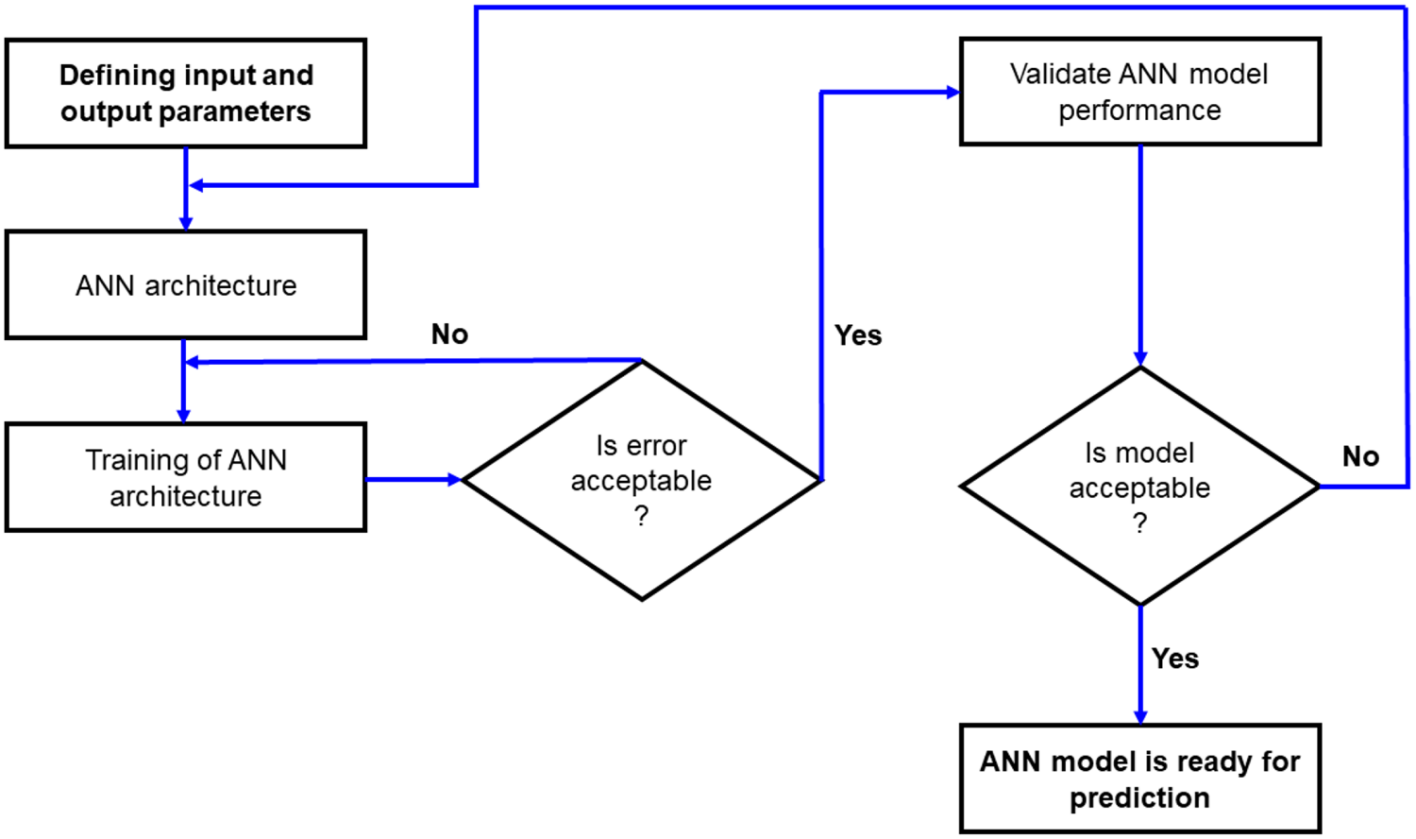
ANN prediction model.

The MATLAB software’s Neural Network Toolbox was used to design and train the network model. In ANN modeling, there are two phases: phase one is to train the network model, and phase two is to validate the network model with new data that was not used for training. Figure 9 depicts the network model’s algorithm.

**Figure 10:**
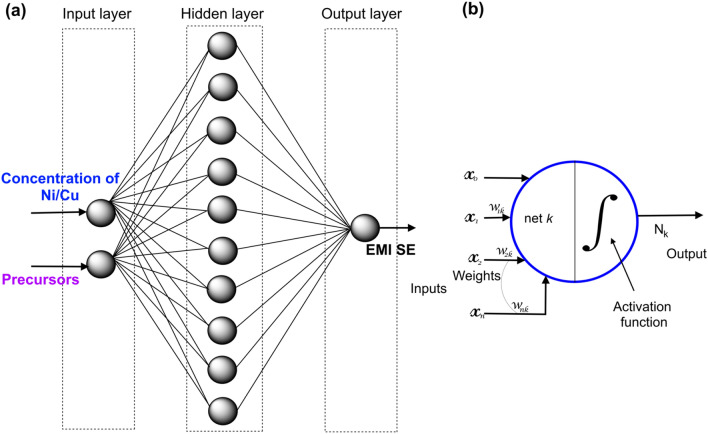
ANN architechure (**a**); architechure of individual neuron (**b**).

One of the most difficult aspects of neural network modeling is determining the best network architecture. As shown in Fig. 10a, a neural network model with back-propagation neural network model (BPNN) is used in this study. The network has three layers: an input layer, a hidden layer, and an output layer. The Ni/Cu concentration and precursor kinds are two inputs, while the EMI shielding effectiveness is one output. In order to identify an optimal solution, several numbers of neurons in the hidden layer were evaluated, and prediction error was computed.

Studies dedicated to the use of artificial neural networks for prediction of EMI shielding were divided into the following stages of implementation:

- Selecting the technical parameters having a significant impact on the quantity of EMI shielding.
- Developing a database for the neural network learning process.
- Creating a set of neural networks and selecting the best of them.
- Calculating a correlation and determining the coefficients.
- Comparing and evaluating an acceptability of the predictive model.

The BPNN is primarily based on an error corrective learning rule. Forward computation and backward learning are the two basic processes in the operation of a neural network since there is no calculation in the input layer of forward computing, the input patterns applied to the neurons are just stimuli to the network. Each neuron in the buried layer determines a net input value depending on its input connection, as shown in Fig. 10b. Nodes are linked because the value of one can affect the value of another. Each connection's weight specifies the relative influence of one node on another.

The net input is calculated by adding the input values together with the weight. The net input is calculated and then transformed into an activation value. The weight on the link between the *ith* neuron in the forward layer and the *kth* neuron is denoted by *w*_*i*k_. The output value of the kth neuron is represented by the Eq. (2):

2 $$N_{k} = \sum\limits_{i = 0}^{n} {w_{ik} x_{k} + x_{0} } ,Z_{k} = f_{a} (N_{k} )$$

where *N*_*k*_ is the linear combination of each of the values multiplied by *w*_*ik*_*, x*_*0*_ is a bias constant, *n* is the number of inputs to the *k*^th^ neuron, and *f*_*a*_ is the activation of neuron *k*. For the prediction of EMI shielding the hidden layer with log-sigmoid (S-shaped curves) activation function is used in this experiment. The log-sigmoid activation function is given in the following Eq. (3):

3 $$Z_{k} = \frac{1}{{1 + \exp ( - N_{k} )}}$$

In the phase of backward learning, the output of the network generated is compared to the desired output, and for each neuron the error is computed. The error between the desired values and the output value of the network is represented as:

4 $$ER = \sum\limits_{k} {E_{k} } = \sum\limits_{k} \frac{1}{2} (T_{k} - Z_{k} )^{2}$$

where *Z*_*k*_ is the *kth* output neuron’s output value and *T*_*k*_ is the intended value of the kth output neuron. Layer after layer, the process of transferring errors backward to the output layer to each neuron in the forward kayer is repeated. To converge the network, each neuron updates the connection weights. This training technique was chosen because to its excellent accuracy and comparable function approximation. Weights and biases are adjusted using the transfer following function:

5 $$\delta w_{ik} = - \frac{1}{{(J^{T} J + \alpha I)}} \times J^{T} \times ER$$

where J is the Jacobian matrix of first derivatives, $\alpha$ is a scalar, and ER is the error function. The input and output parameters of the data sets in their original form vary widely, making neural networks unsuitable for training. Furthermore, in order to create a viable neuron, the input parameters must be pre-processed by normalizing and transforming them. The normalized value *(N*_*i*_*)* was determined for each raw input and output data set *(d*_*i*_*)* as follows:

6 $$N_{i} = \frac{2}{{r_{\max } - r_{\min } }}(r_{i} - r_{\min } ) - 1$$

where *r*_*max*_ and *r*_*min*_ are the raw data's maximum and lowest values, respectively.

The back-propagation neural networks model was trained using data sets from laboratory experiments. The data is separated into three sections at random: training data, testing data, and validation data. The neural network training setup was designed and developed. To find the best network design, the number of neurons in the hidden layer is determined. To measure the network’s correctness, the mean squared error *(MSE)* and the coefficient of multiple determinations, *R*^2^, are calculated.

### Characterization

#### Surface properties

The scanning electron microscopy by Tescon-Vega was used to study the surface morphology of conductive fabric before and after silanization treatment. The acceleration voltage of 20 kV was used during the measurement.

#### Thermogravimetric analysis (TGA)

In this work, the thermal properties ultra-light weight Ni/Cu coated polyester fibrous materials was analyzed by using of TGA-4000 from Perkin Elmer, Germany. Thermal stability and weight loss vs temperature were measured in 5 mg samples under controlled heating and environmental settings in the air and inert nitrogen atmosphere. The analysis can be done in inert (nitrogen) atmosphere under following conditions: flow rate of 50 mL/min and a heating rate of 20 °C/min over a temperature range of 50–700 °C.

#### Measurement of EM SE

**Figure 11:**
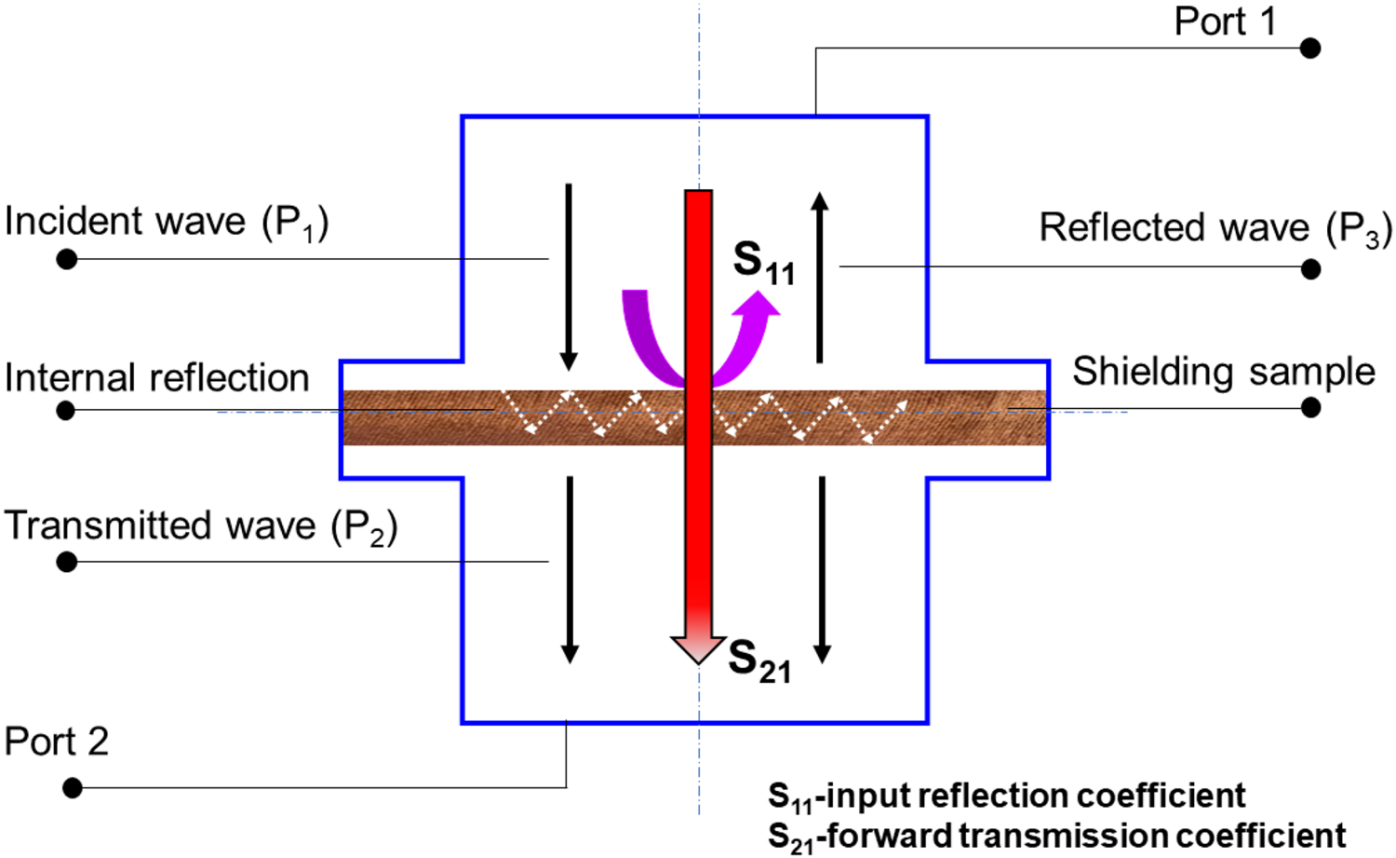
Measurement of SE for ultra-light Ni/Cu coated polyester fibrous materials.

The conductive fabric has been analysed their electromagnetic shielding effectiveness (EMSE) by simple EMSE tester (i.e., 30 MHz to 1.5 GHz) which has been already well described^31^ in the accordance of ASTM D4935-10^32^. The standard room temperature and relative humidity was maintained during the EMSE measurement. The set-up of the instrument was well explained in the previous research manuscripts from our laboratory^31,33–35^. Figure 11 described the testing principles of EMSE measurement. Figure S2 shows the EM SE measurement of Ni/Cu coated ultra-light weight Ni/Cu coated polyester fibrous materials. The SE can be interpreted by the forward transmission coefficient S_21_, which is the ratio of power without and with shielding material, the calculation method is as following Eq. (7):

7 $$SE(S21) = - 10\log \left( {\frac{{P_{1} }}{{P_{2} }}} \right) = 10\log \left( {\frac{{P_{2} }}{{P_{1} }}} \right)$$

where P_2_ and P_1_ are the powers received with and without the fabric presence respectively. Sample reflects the EM wave signal from the transmitting antenna is interpreted as the EM reflection coefficient and received by port 1. The input reflection coefficient is calculated as the ratio of receiving reflected power P_3_ and the received power without the fabric present P_1_ is given by the Eq. (8),

8 $$S_{11} = 10\log \left( {\frac{{P_{3} }}{{P_{1} }}} \right)$$

The measurements were performed under the following laboratory conditions: T = 23.9 °C, RH = 48%. Each sample was measured five times at different locations.

### Analytical investigation

#### Correlation coefficient

Pearson correlation coefficients r are used to evaluate the matching between the predicted and experimental values of *I*_*1*_ and *I*_*2*_ variables. The level of linear relationship between the variables are determined using Pearson correlation coefficient which takes the values from range [− 1; 1]. The dependence is said to be positive when the values are nearer to 1 and negative when it is nearer to − 1 and no linear dependence when value is 0. Equation (9) represents the relationship allowing the calculation of values of coefficients r

9 $$r = \frac{{\sum\nolimits_{i = 1}^{n} {(E_{i} - \overline{E})(P_{i} - \overline{P})} }}{{\sqrt {\sum\nolimits_{i = 1}^{n} {(E_{i} - \overline{E})^{2} } \sum\nolimits_{i = 1}^{n} {(P_{i} - \overline{P})^{2} } } }}$$

where, *E*_*i*_ experimental value for *i*, *P*_*i*_ predicted value for *i* and *n* number of records.

#### Prediction errors

ANN substantial is implemented as an element of analyzing results to predict the EMI shielding to evaluate the admissibility of the predictions resulted by a given predictive model. The experimental values are compared to the predicted values in MSE and calculated indicates the error in the units (or squared units), aiding result analysis. A perfect fit is achieved when MSE values must be zero. The accuracy of the model is analyzed using post errors. Most predominantly used prediction error is represented below as

10 $$MSE = \frac{1}{n}\sum\limits_{i = 1}^{n} {(E_{i} - P_{i} )^{2} }$$

11 $$MAPE = \frac{1}{n}\sum\limits_{i = 1}^{n} {\left| {\frac{{E_{i} - P_{i} }}{{E_{i} }}} \right|}$$

## Supplementary Information

Supplementary Information.

## Data Availability

The data that support the findings of this study are available on request from the corresponding author (APP). The data are not publicly available due to the privacy.
