# Supplementary material for: Neural network model applied to electromagnetic shielding effectiveness of ultra-light Ni/Cu coated polyester fibrous materials
Source: Sci Rep. 2022 May 21;12:8609. doi: 10.1038/s41598-022-12593-8 (PMC9124190; doi:10.1038/s41598-022-12593-8)
Supplement: Supplementary file 1 — Supplementary Information. [file 41598_2022_12593_MOESM1_ESM.docx]

**Supporting Information**

***Neural network model applied to electromagnetic shielding effectiveness of ultra-light Ni/Cu coated polyester fibrous materials***

*Aravin Prince Periyasamy^1,3*^, Lekha Priya Muthusamy^2^, Jiri Militky^3*^*

*^1^Department of Bioproducts and Biosystems, School of Chemical Engineering, Aalto University, Espoo, Finland.*

*^2^Department of Mathematics, Government arts college, Coimbatore, India.*

*^3^Department of Material Engineering, Faculty of Textile Engineering, Technical University of Liberec, Studentska 2, 46117, Czech Republic.*

**EMI Measurement**

Electromagnetic shielding effectiveness (EM SE) of Ni/Cu coated fabric was analyzed by using a device described in Veronika et al, [1,2] in the accordance of ASTM D4935-10 (*ASTM D 4935-10:2010: Standard Test Method for Measuring the Electromagnetic Effectiveness of Planar Materials*, 2010). During the measurement, controlled temperature and relative humidity was maintained (27.1 °C & 41 % RH). EM SE of nickel coated sample was measured over frequency range of 30 MHz to 1.5 GHz and the results were expressed in decibels (dB). The set-up consists of a sample holder with its input and output connected to the network analyzer. A shielding effectiveness test fixture (Electro-Metrics, Inc., model EM-2107A) was used to hold the sample. The design and dimension of sample holder follows the ASTM method mentioned above. Network analyzer Rohde & Schwarz ZN3 was used to generate and receive the electromagnetic signals. The standard mentioned above determines the electromagnetic shielding effectiveness of the fabric using the insertion-loss method. The set-up of the instrument was well explained in the previous research manuscripts from our laboratory [1,2]. The set-up consisting of the distribution of electrical and magnetic fields are in the coaxial transmission. Before EM SE measurements, the instrument was calibrated. Ten repetitive measurements were conducted, and the average values used to plot the graphs.

### ***Scanning electron microscope with EDS***

The scanning electron microscopy by Tescon-Vega was used to study the surface morphology of conductive fabric before and after silanization treatment. The acceleration voltage of 20 kV was used during the measurement. It is equipped with energy-dispersive X-ray spectroscopy (EDS, FEI), for surface analysis of Ni/Cu plated fabric.

Our aim is to assess the homogeneous distribution of coatings, five recurrent measurements were carried out on different parts of Ni/Cu plated PET fabrics, average values of composition of elements. Figure S1 displays the documented information of EDS from Ni/Cu plated PET fabrics. Strong spectra demonstrated together with certain elements like copper, nickel, titanium, carbon, and oxygen, confirms the presence of various elements.


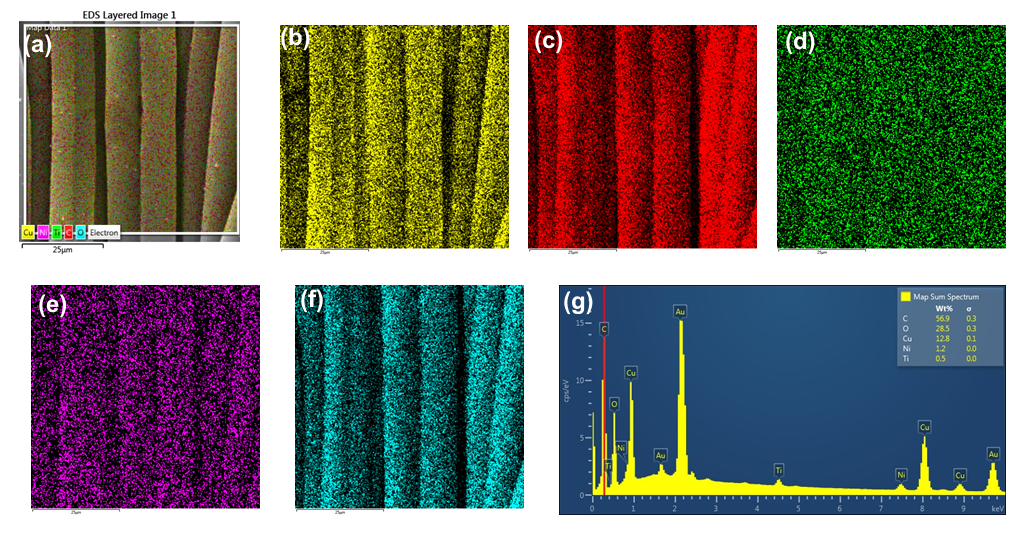


Figure S1. EDS characterization of Ni/Cu fabric with distribution of different atoms on the fabric (a), distribution of Cu (b), distribution of Ni (c), distribution of Ti (d), distribution of C (e), distribution of O (f) weight percentage of each atom (g).


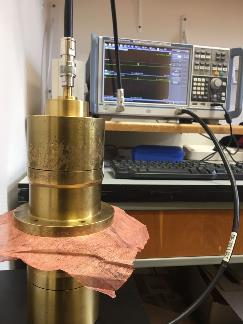


Figure S2*.* Ni/Cu samples during the EMI sheidling measurement.

**Reference**

[1] V. Šafářová, J. Militký, Electromagnetic shielding properties of woven fabrics made from high-performance fibers, Textile Research Journal. 84 (2014) 1255–1267. https://doi.org/10.1177/0040517514521118.

[2] V. Šafářová, M. Tunák, J. Militký, Prediction of hybrid woven fabric electromagnetic shielding effectiveness, Textile Research Journal. 85 (2015) 673–686. https://doi.org/10.1177/0040517514555802.
